# Promising biotherapeutic prospects of different probiotics and their derived postbiotic metabolites: in-vitro and histopathological investigation

**DOI:** 10.1186/s12866-023-02866-1

**Published:** 2023-05-03

**Authors:** Mona S. El Far, Azza S. Zakaria, Mervat A. Kassem, Abdalla Wedn, Maha Guimei, Eva A. Edward

**Affiliations:** 1 Department of Microbiology and Immunology, Faculty of Pharmacy, Alexandria University, Alexandria, Egypt; 2 Department of Pharmacology and Toxicology, Faculty of Pharmacy, Alexandria University, Alexandria, Egypt; 3 Department of Pathology, Faculty of Medicine, Alexandria University, Alexandria, Egypt

**Keywords:** Probiotics, Cell free supernatant, Resistance genes, Antimicrobial, Antibiofilm, Anti-inflammatory, Crystal violet assay, Carrageenan-induced rat paw edema model, Histopathological examination

## Abstract

**Background:**

Probiotics and their derived postbiotics, as cell-free supernatants (CFS), are gaining a solid reputation owing to their prodigious health-promoting effects. Probiotics play a valuable role in the alleviation of various diseases among which are infectious diseases and inflammatory disorders. In this study, three probiotic strains, *Lactiplantibacillus plantarum*, *Lacticaseibacillus rhamnosus*, and *Pediococcus acidilactici*, were isolated from marketed dietary supplements. The antimicrobial activity of the isolated probiotic strains as well as their CFS was investigated. The neutralized CFS of the isolated probiotics were tested for their antibiofilm potential. The anti-inflammatory activity of the isolated *Lactobacillus* spp., together with their CFS, was studied in the carrageenan-induced rat paw edema model in male Wistar rats. To the best of our knowledge, such a model was not previously experimented to evaluate the anti-inflammatory activity of the CFS of probiotics. The histopathological investigation was implemented to assess the anti-inflammatory prospect of the isolated *L. plantarum* and *L. rhamnosus* strains as well as their CFS.

**Results:**

The whole viable probiotics and their CFS showed variable growth inhibition of the tested indicator strains using the agar overlay method and the microtiter plate assay, respectively. When tested for virulence factors, the probiotic strains were non-hemolytic lacking both deoxyribonuclease and gelatinase enzymes. However, five antibiotic resistance genes, *bla*Z, *erm*B, *aac(6’)- aph(2”), aph(3’’)-III*, and *van*X, were detected in all isolates. The neutralized CFS of the isolated probiotics exhibited an antibiofilm effect as assessed by the crystal violet assay. This effect was manifested by hindering the biofilm formation of the tested *Staphylococcus aureus* and *Pseudomonas aeruginosa* clinical isolates in addition to *P. aeruginosa* PAO1 strain. Generally, the cell cultures of the two tested probiotics moderately suppressed the acute inflammation induced by carrageenan compared to indomethacin. Additionally, the studied CFS relatively reduced the inflammatory changes compared to the inflammation control group but less than that observed in the case of the probiotic cultures treated groups.

**Conclusions:**

The tested probiotics, along with their CFS, showed promising antimicrobial and anti-inflammatory activities. Thus, their safety and their potential use as biotherapeutics for bacterial infections and inflammatory conditions are worthy of further investigation.

**Supplementary Information:**

The online version contains supplementary material available at 10.1186/s12866-023-02866-1.

## Background

Probiotics are gaining a solid reputation owing to their prodigious health-promoting effects which are rigorously investigated through scientific research that highlights their precious benefits [1]. A wide array of genera comprises strains that may be qualified as probiotics; the *Bifidobacterium* genus as well as the lactic acid bacteria (LAB) genera, particularly the *Lactobacillus* genus, are the most common [2].

Probiotics play a valuable role in the alleviation of infectious diseases, diarrheal disorders, inflammatory conditions, lactose intolerance, allergies, and colorectal cancer [3]. The underlying mechanisms of probiotics’ diverse health-promoting effects include strengthening of the gut mucosal barrier, enhanced adhesion to intestinal cells, competitive exclusion of different pathogens, secretion of antimicrobial compounds, and immune response modulation [4].

Probiotic bacteria produce a myriad of antimicrobial substances such as hydrogen peroxide, bacteriocins, biosurfactants, and organic acids that can hinder the colonization of pathogens [5]. Moreover, various metabolites secreted by probiotics are powerful antibiofilm agents that could impede biofilm formation or disperse pathogens’ preformed biofilms [6]. In vitro as well as in vivo models revealed the inflammatory regulation mechanisms exerted by several probiotic strains, particularly *Lactobacillus* spp. Their immunomodulatory properties have been attributed to the reduction of inflammatory responses through different immune cells among which are natural killer cells, B-lymphocytes, T-lymphocytes, and macrophages [7, 8].

Despite their tremendous benefits, safety concerns of probiotics should be thoroughly evaluated including the risk of transferability of antibiotic resistance or virulence genes from probiotics to other bacterial species, the risk of opportunistic infections, and the potential detrimental metabolic activities by probiotics that could pose a harmful impact on the host [9].

Owing to the rising global tendency to natural non-drug approaches for health improvement, the probiotic market has flourished rapidly and it is expected to continue growing in the coming years [10, 11]. Probiotic dietary supplements commonly consist of millions to billions of probiotic bacteria packed in one capsule or tablet. Dietary supplements incorporating such high counts of probiotic bacteria impose an inevitable threat that might exaggerate the antibiotic resistance problem. Such probiotic strains could act as reservoir organisms for antibiotic resistance determinants that might spread to other intestinal microflora and pathogenic microbes which share the same residence in the human gut [11].

Recently, new probiotic-related concepts, such as postbiotics and parabiotics, have evolved and are expected to cause a radical medical improvement in the world of microbial biotherapy. Postbiotics include the CFS of probiotics with numerous soluble factors such as proteins, organic acids, and short-chain fatty acids. Parabiotics comprise microbial components such as teichoic acids, exopolysaccharides, and cell surface proteins. There is an increasingly growing trend to use probiotic-derived components as they are thought to be safer, more stable, and more specific in action than the whole viable probiotics. One of their advantages over the whole viable cells is that there would be no chance of transfer of antibiotic resistance or virulence genes among bacterial species [6]. Nonetheless, further investigations are needed to prove their efficacy as novel biotherapeutic agents.

This study aimed to investigate the antimicrobial and antibiofilm activities of probiotic strains, focusing on their CFS, isolated from three commercially available dietary supplements. Moreover, attempts were made to assess the anti-inflammatory activity of the isolated *Lactobacillus* spp. probiotic strains, as well as their CFS, using the carrageenan-induced rat paw edema model in male Wistar rats.

## Results

### The identification of the collected isolates by MALDI-TOF MS

Using MALDI-TOF MS, the three strains isolated from dietary supplements were identified as follows: P3: *Lactiplantibacillus plantarum*, P4: *Lacticaseibacillus rhamnosus*, and P5: *Pediococcus acidilactici* (Table 1).

**Table 1:** Information on the studied dietary supplements and their probiotic strains

Dietary supplement code	Manufacturer	Dosage form	Isolated strain code	Probiotic strain*
DSP3	Nature’s Bounty®	Tablet	P3	*Lactiplantibacillus plantarum*
DSP4	Ther-Biotic®	Capsule	P4	*Lacticaseibacillus rhamnosus*
DSP5	Nutrilots®	Capsule	P5	*Pediococcus acidilactici*

* identified by MALDI-TOF MS

### Antimicrobial activity of probiotic strains

**Fig. 1:**
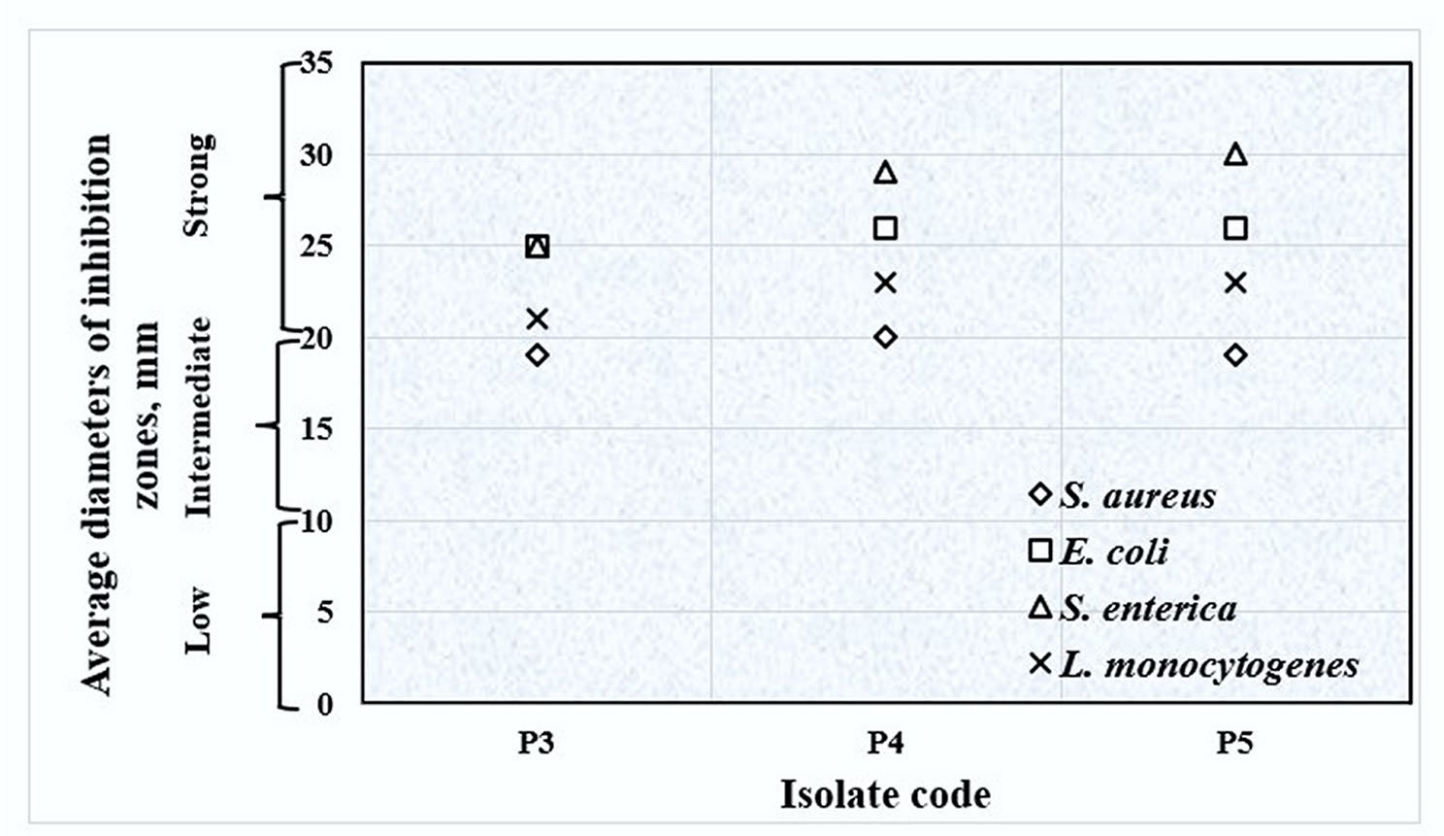
Antibacterial activity of the isolated probiotic strains against indicator strains using the agar overlay technique

Based on the agar overlay technique, the isolated probiotics strongly inhibited the growth of *Listeria monocytogenes* EGD-e (serotype 1/2a), *Escherichia coli* NCTC 10418, and *Salmonella enterica subsp. enterica serovar* Typhimurium ATCC 14028 indicator strains. Moderate growth inhibition was noticed in the case of *Staphylococcus aureus* ATCC 6538. None of the examined strains showed any inhibitory activity against *Candida albicans* ATCC 10231 (Fig. 1).

### Characterization of selected virulence factors and antimicrobial susceptibility testing

The tested strains were non-hemolytic lacking both deoxyribonuclease and gelatinase enzymes. They showed resistance to kanamycin, gentamicin, and vancomycin with MIC ranges of (1024 - >1024 µg/mL), (64 - >512 µg/mL), and (> 256 µg/mL), respectively. Only P4 showed susceptibility towards ampicillin (MIC = 1 µg/mL). P3 and P4 were sensitive to erythromycin with MIC values of 1 and 0.5 µg/mL, respectively. Five, out of six, tested antibiotic resistance genes, *bla*Z, *erm*B, *aac(6’)- aph(2”), aph(3’’)-III, and van*X, were detected in all isolates. However, *bla* gene was not found at all (Table 2).

**Table 2:** Minimum inhibitory concentrations of some antibiotics against probiotic strains and the detected antibiotic resistance genes

Antibiotic	Genes of resistance	Probiotic strain MIC^a^ (µg/mL), Antibiotic resistance pattern		
		*Lactiplantibacillus plantarum* (P3)	*Lacticaseibacillus rhamnosus* (P4)	*Pediococcus acidilactici* (P5)
		4, R	1, S	8, R
Ampicillin	*bla*	**-**	**-**	**-**
	*bla*Z	**+**	**+**	**+**
Kanamycin		˃1024, R	1024, R	˃1024, R
	*aph(3’’)-III*	**+**	**+**	**+**
Gentamicin		512, R	64, R	˃512, R
	*aac(6’)-aph(2’’)*	**+**	**+**	**+**
Vancomycin		> 256, R	> 256, R	> 256, R
	*van*X	**+**	**+**	**+**
Erythromycin		1, S	0.5, S	4, R
	*erm*B	**+**	**+**	**+**

^a^The MICs are interpreted according to the European food safety authority (EFSA) 2018. S: sensitive, R: resistant

### The mechanism of the antimicrobial activity of the tested probiotic strains

**Fig. 2:**
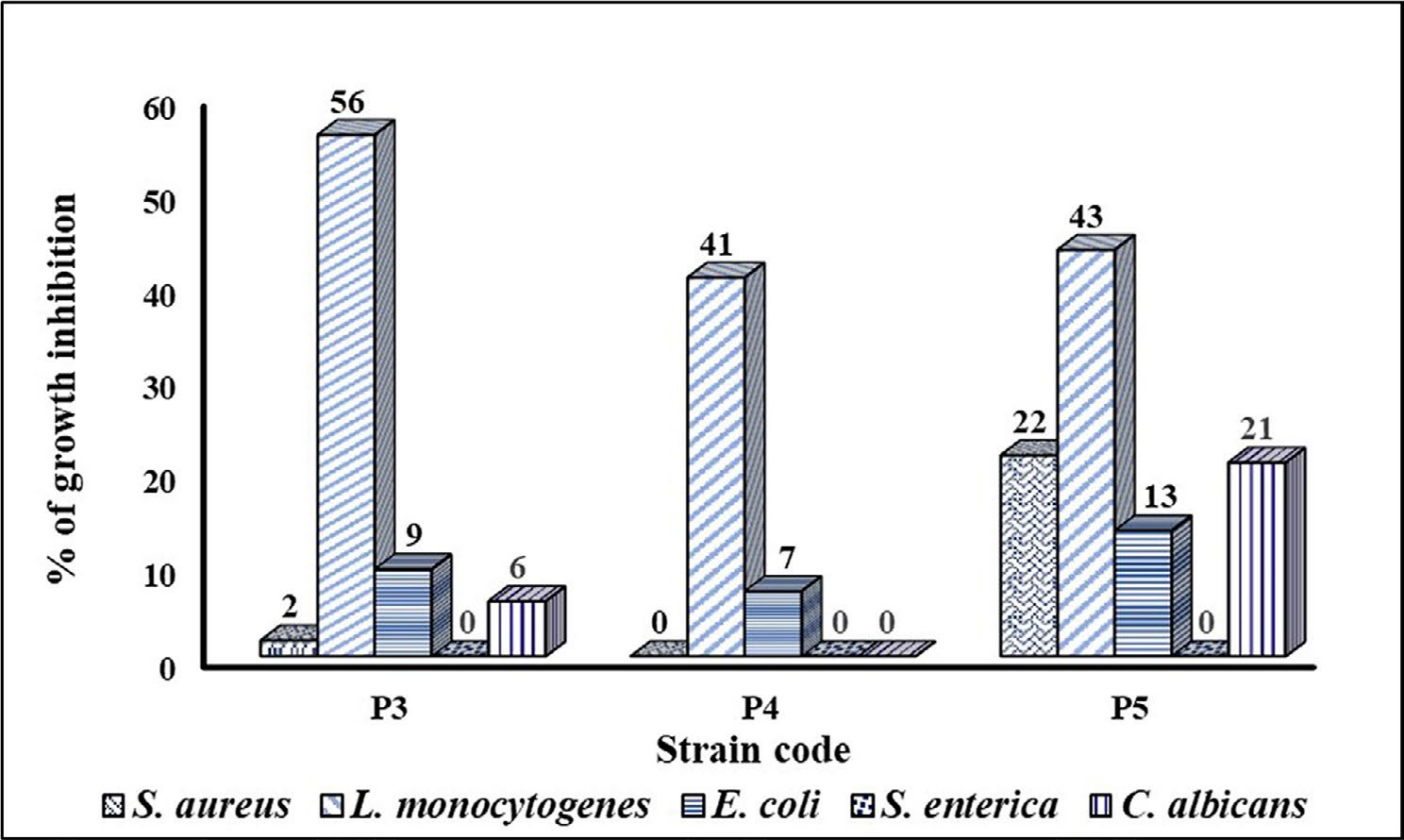
Comparative antimicrobial activity of the nCFS of the isolated probiotic strains against selected Gram-positive, Gram-negative, and *C. albicans* standard strains

The probiotics’ non-neutralized CFS showed higher than 89% growth inhibition of the standard Gram-positive and Gram-negative strains. Similarly, a noticeable antifungal activity was noticed (about 56.5–68% inhibition of *C. albicans* growth). On the contrary, upon neutralization of the CFS, the antimicrobial activity was reduced or completely abolished. The antibacterial activity of the nCFS of P4 was eliminated against *S. aureus* while the percentage of growth inhibition of *S. aureus* by the nCFS of P3 and P5 was drastically reduced to 2% and 22%, respectively. Against *L. monocytogenes*, the nCFS of the tested probiotics showed relatively higher inhibitory activity, compared to *S. aureus*, with percentages of growth inhibition ranging between 41% (in the case of P4) and 56% (in the case of P3). The neutralization of the CFS resulted in extremely lower percentages of growth inhibition of *E. coli* ranging between 7 and 13% while the antibacterial activity was completely abolished against *S. enterica.* In the case of *C. albicans*, the percentages of growth inhibition by the nCFS of P3 and P5 were reduced to 6% and 21%, respectively, while no inhibitory activity was detected in the case of P4 (Fig. 2).

### The effect of the nCFS on the biofilm formation of selected pathogenic strains

**Fig. 3:**
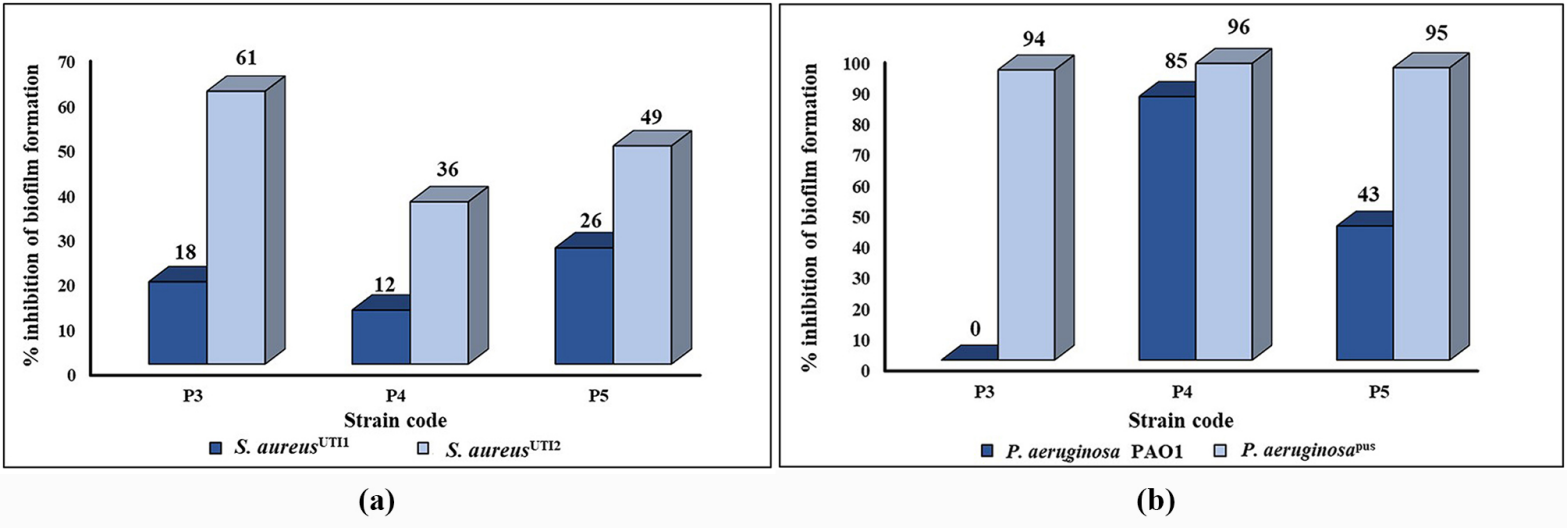
Effect of the nCFS of the tested probiotic strains on the biofilm formation of: (**a**) *S. aureus* clinical isolates, and (**b**): *P. aeruginosa* PAO1 standard strain and *P. aeruginosa*^pus^ clinical isolate

The nCFS of the tested probiotics hindered the biofilm formation of the 2 tested *S. aureus* clinical isolates but to different extents. The percentages of inhibition of biofilm formation were higher in the case of *S. aureus*^UTI2^ (61%, 36%, and 49% in the case of P3, P4, and P5, respectively) compared to *S. aureus*^UTI1^ (18%, 12%, and 26% in case of P3, P4, and P5, respectively) (Fig. 3a). Regarding pseudomonal biofilms, a remarkable inhibitory activity of the biofilm formation of *P. aeruginosa*^pus^ clinical isolate was observed for all the examined probiotics (more than 90% inhibition). Additionally, the nCFS of P4 and P5 showed 85% and 43% inhibition of biofilm formation of *P. aeruginosa* PAO1 strain, respectively. Despite its profound inhibitory activity against biofilm formation in *P. aeruginosa*^pus^ clinical isolate, the nCFS of P3 completely failed to inhibit the biofilm formation of *P. aeruginosa* PAO1 strain (Fig. 3b).

### Anti-inflammatory activity of *Lactobacillus* spp. probiotic strains in male Wistar rats

**Fig. 4:**
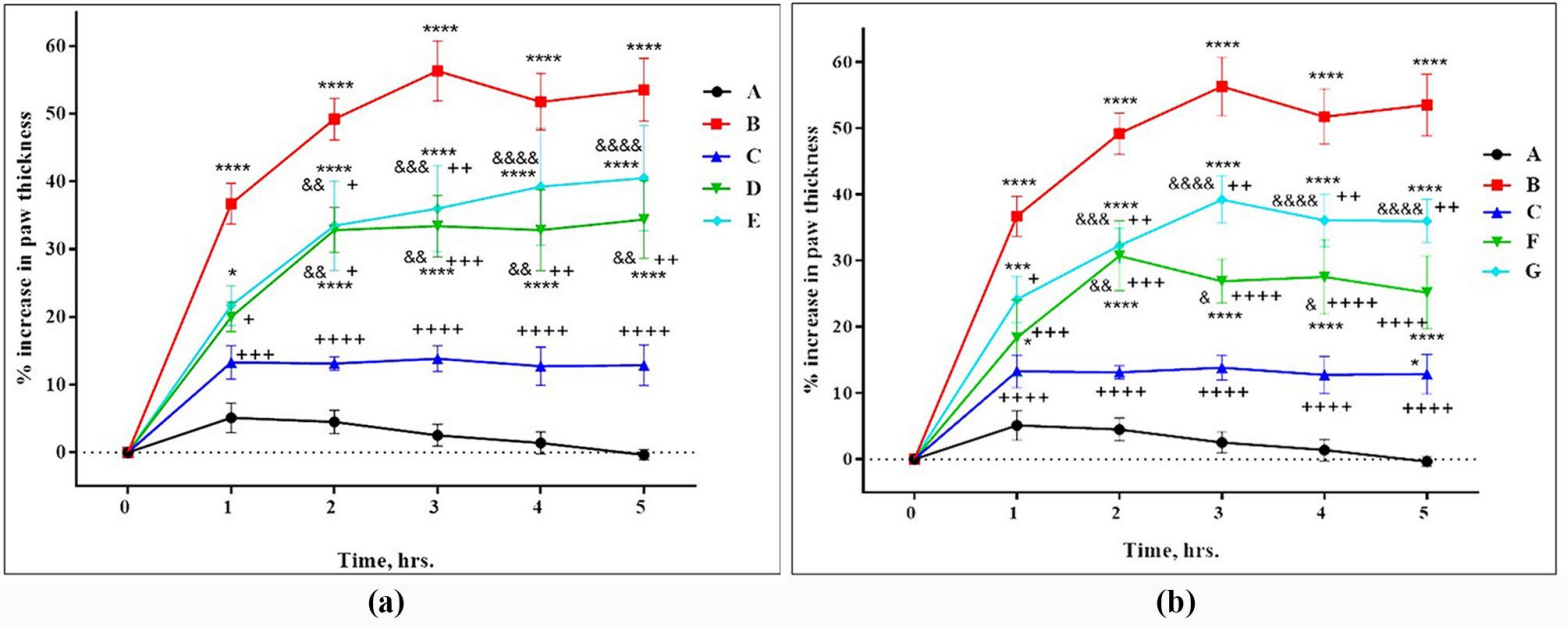
Percentage increase in the paw thickness of Wistar rats in different groups. (**a**) Groups: A: saline control group, B: carrageenan control group, C: indomethacin + carrageenan group, D: *L. rhamnosus* culture + carrageenan group, and E: *L. rhamnosus* CFS + carrageenan group. (**b**): Groups: A: saline control group, B: carrageenan control group, C: indomethacin + carrageenan group, F: *L. plantarum* culture + carrageenan group, and G: *L. plantarum* CFS + carrageenan group. Data expressed as mean ± SEM of n = 6 rats/group (significant at P < 0.05). * P < 0.05 VS. saline control group. + P < 0.05 VS. carrageenan control group. & P < 0.05 VS. indomethacin + carrageenan group. * or + or & P < 0.05, ** or + + or && P < 0.01, *** or +++ or &&& P < 0.001, **** or ++++ or &&&& P < 0.0001

At 1 h post saline injection, the paw thickness of the rats in Group A (saline control group) did not significantly change, compared to 0 h, with the least percentages increase in paw thickness that did not exceed 5.12% ± 2.19. Then, it declined to 4.51% ± 1.73, 2.55% ± 1.61, 1.4% ± 1.61, and 0% ± 0.77 at 2, 3, 4, and 5 h intervals, respectively. On the contrary, Group B (carrageenan control group) exhibited the highest increase in paw thickness with progressive edema that was significantly different from the saline control group throughout the time intervals (significant at P < 0.0001). Group C (indomethacin + carrageenan group) showed the highest anti-inflammatory action, post-carrageenan injection, with a minimum percentage increase in the paw thickness that was remarkably lower than the carrageenan control group (significant at P < 0.0001). (Fig. 4a and b).

*L. rhamnosus* culture in group D (*L. rhamnosus* culture + carrageenan) moderately reduced the edema resulting from carrageenan compared to indomethacin. The increase in the paw thickness, observed in group D, was significantly lower relative to the inflammation control group throughout the time intervals with the highest statistical significance (P < 0.001) noticed at 3 h interval. Regarding *L. rhamnosus* CFS, it had a relatively weak impact on reducing the paw swelling caused by carrageenan in group E (treated with *L. rhamnosus* CFS) compared to indomethacin, the standard anti-inflammatory drug. The paw thickness increased by 21.71% ± 2.93, one-hour post-carrageenan injection, without a considerable difference from indomethacin. Moreover, at 2 and 3 h intervals, there was a statistically significant difference from the inflammation control group at P < 0.05 and P < 0.01, respectively. However, the paw thickness continued to increase with 39.26% ± 8.63, and 40.51% ± 7.78 at 4 and 5 h intervals, respectively, with no statistically significant difference from the carrageenan control group (Fig. 4a**).**

On the other hand, group F (treated with *L. plantarum* culture) showed an 18.37% ± 5.71 and 30.74% ± 5.27 increase in the paw thickness at 1 and 2 h intervals, respectively, compared to 0 h. The increase in the paw thickness started to decline after the second hour with a statistically significant difference at P < 0.0001 from the carrageenan control group noticed at 3, 4, and 5 h intervals. Regarding group G (treated with *L. plantarum* CFS), the *L. plantarum* CFS was able to reduce the paw edema caused by carrageenan with a statistically significant difference, ranging between P < 0.05 and P < 0.01, noticed at all time intervals relative to the inflammation control group (Fig. 4b).

### Histopathological evaluation of paw tissue sections using hematoxylin and eosin staining

**Fig. 5:**
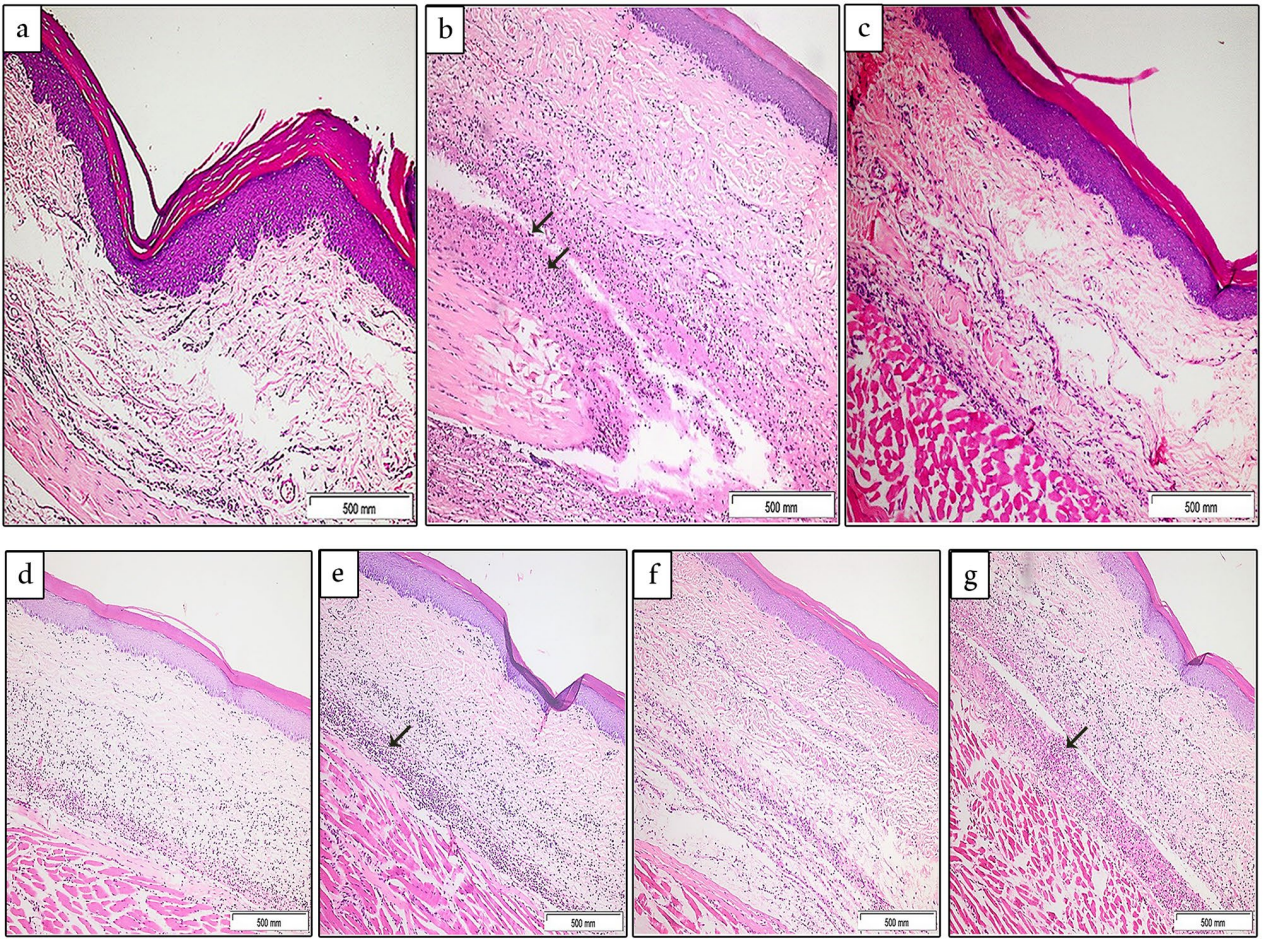
Inflammatory features in untreated and treated rat groups: (**a**) Sections in the rat paw tissue from group A showing almost no elicited inflammation. (**b**) Sections from group B showing significant edema and intense neutrophilic infiltrates (arrows) in the dermis, 5 h after the sub-plantar injection of carrageenan. (**c**) Sections from the indomethacin-treated group showing significant improvement in the inflammatory features compared to group B. (**d**, **f**) The probiotic culture-treated groups; (group D and group F) both showing a moderate anti-inflammatory response with a moderate number of inflammatory cellular infiltrates and edema seen in the deep dermis when compared to group B. (**e**, **g**) Sections from the probiotics CFS treated groups; Group E and group G showing mild improvement with dense inflammatory cells relative to the probiotic culture-treated groups (arrows), yet also showing a statistically significant difference (P < 0.001) when compared to the inflammation control group B

The sub-plantar injection of carrageenan (1%) into the rats’ right hind paw elicited a significant inflammatory response in the form of a dense acute inflammatory cellular infiltration rich in neutrophils, severe edema, severe congestion as well as tissue necrosis observed in the superficial and deep dermal tissues. Moreover, inflammatory cells were also noted to infiltrate the underlying muscle tissue. These changes were demonstrated in the untreated group (group B: carrageenan control group) with a statistically significant difference in the average total histological scores (P < 0.0001) when compared to group A (saline control group) which showed only mild inflammatory changes upon saline injection (Fig. 5a and b).

On the other hand, examination of tissue sections from the treated groups showed that the indomethacin-treated group (group C) demonstrated the most significant improvement of the inflammatory response, compared to the inflammation control group (P < 0.0001), with only minimal inflammatory cell infiltrate (score + 1), mild edema (score + 1) and moderate congestion (score + 2) being noted (Fig. 5c).

When comparing the probiotic-treated groups, the *L. rhamnosus* culture-treated group (group D) showed moderate inflammation (score + 2) and minimal edema (score + 1) together with mild congestion (score + 1) and a considerable decline in the number of inflammatory cells compared to the untreated group B (Fig. 5d). The average total histological score in group D was statistically significant at P < 0.0001 compared to the carrageenan control group. Whereas in group E (*L. rhamnosus* CFS + carrageenan) the number of inflammatory cells, congestion, and edema were more evident compared to group D, with histological scores + 2 for each, but were still less intense than those seen in group B with a statistically significant difference being noted (P < 0.001) (Fig. 5e).

Regarding *L. plantarum* culture-treated group (group F), a mild to moderate amount of inflammatory cell infiltration (score + 2) and mild edema (score + 1) were detected (Fig. 5f). The average total histological scores were significantly lower than that manifested in group B (P < 0.0001). On the other hand, the anti-inflammatory effect was less noted in group G (*L. plantarum* CFS + carrageenan) with denser neutrophilic infiltration (score + 2) and a higher degree of edema in the deep dermis (score + 2) compared to group F. Yet, those changes were still milder than those revealed in the carrageenan control group (P < 0.001) (Fig. 5g).

**Fig. 6:**
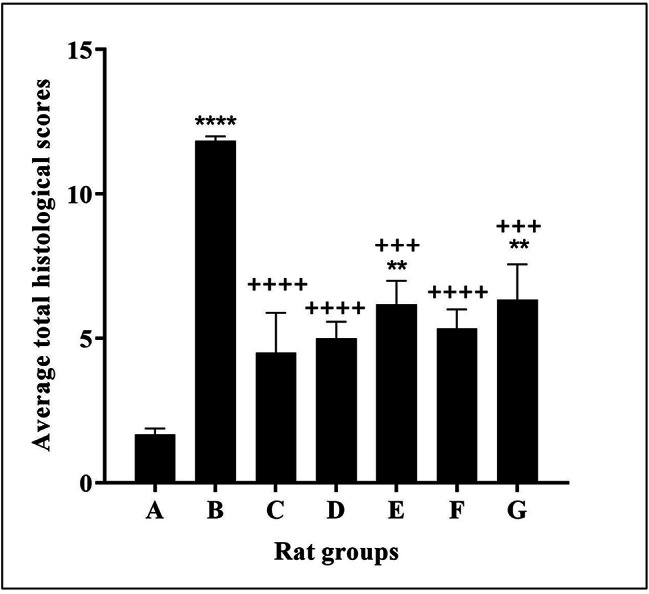
The average total histological scores of congestion, inflammation, edema, and necrosis observed in the examined paw tissue sections of the 6 rats in each group. * P < 0.05 VS. saline control group. + P < 0.05 vs. carrageenan control group. * or + P < 0.05, ** or + + P < 0.01, *** or +++ P < 0.001, and **** or ++++ P < 0.0001

The average total histological scores of congestion, inflammation, edema, and necrosis observed in the examined paw tissue sections of the 6 rats in each group were calculated. There was an apparent improvement in the inflammatory changes in all treated groups compared to the carrageenan control group. The indomethacin-treated group showed the most significant anti-inflammatory effect with the most improvement in the average total histological scores compared to the inflammation control group (P < 0.0001). The probiotic culture-treated groups (D and F) also significantly reduced these inflammatory changes (P < 0.0001). The probiotic CFS-treated groups (E and G) displayed less satisfactory scores compared to their respective probiotics cultures groups. However, they still showed a significant anti-inflammatory effect when compared to the inflammation control group (P < 0.001) (Fig. 6).

## Discussion

The past few decades have witnessed an unceasing consumption of probiotic products. The global probiotics market size is assumed to be rising at an estimated rate of 7% per year [12] and is expected to reach about 69.3 billion dollars by 2023 [13]. In the present study, 3 LAB strains: *Lactiplantibacillus plantarum* (P3), *Lacticaseibacillus rhamnosus* (P4), and *Pediococcus acidilactici* (P5) were isolated from marketed dietary supplements.

The antagonistic activity against different pathogens is considered one of the imperative selection criteria for probiotics. The inhibitory action of LAB is commonly attributed to secreted antimicrobial metabolites, including organic acids, H_2_O_2_, bacteriocins, and biosurfactants [14], which are crucial for the powerful competitive exclusion of pathogens in the GIT and the establishment of a probiotic benefit to the host [15]. The studied LAB isolates showed inhibitory activity against the tested Gram-positive and Gram-negative indicator bacterial strains. Similarly, the inhibitory activity of various *Lactobacillus* spp. against a broad range of pathogens, among which were *E. coli, S. enterica, S. aureus*, and *L. monocytogenes*, has been previously reported [16–19]. Khalkhali et al. demonstrated a broad-spectrum antibacterial activity of *P. acidilactici* strains against *L. monocytogenes, S. aureus, E. coli*, and *S. typhi* [20].

In line with our findings, the absence of important virulence determinants, such as gelatinase, DNase, and hemolytic activities, was reported among strains belonging to *Lactobacillus* and *Pediococcus* spp. which encourages their safety as probiotics [21, 22].

The assessment of the antibiotic resistance profile of LAB strains is a key criterion for their safety evaluation as probiotics. In our study, the examined probiotics were resistant to kanamycin, gentamicin, and vancomycin. However, they showed some discrepancies in their sensitivity towards ampicillin and erythromycin antibiotics. In general, *Lactobacillus* spp. show high resistance to aminoglycosides [23]. Aminoglycoside resistance was also considered an intrinsic property among different *Pediococcus* spp. [24]. *Pediococcus* spp., in addition to most of the *Lactobacillus* spp. including *L. plantarum*, and *L. rhamnosus*, are intrinsically resistant to glycopeptides due to the absence of D-Ala–D-Ala dipeptide which is the antibiotic’s target [23, 25]. On the other hand, the susceptibility of lactobacilli and pediococci to ampicillin and erythromycin is commonly reported in the literature [23, 24]. Nonetheless, based on our findings, ampicillin resistance was detected in P3 and P5, and erythromycin resistance was observed in P5. Previous studies have reported a phenotypic resistance to penicillins such as ampicillin and erythromycin among some strains of *Pediococcus* and *Lactobacillus* spp. [24, 26].

In the present study, the five tested antibiotic resistance genes, *aph(3’’)-III, aac(6’)- aph(2”), van*X, *erm*B, and *bla*Z, were detected in the tested isolates. On the contrary, *bla* gene was not detected at all. Matching with our findings, Hummel et al. have previously noticed the absence of *bla* gene in phenotypically resistant *Lactobacillus* and *Pediococcus* isolates [27].

The presence of *van*X, the gene encoding for D-alanyl-D-alanine dipeptidase, has been frequently reported in *Lactobacillus* spp. [28]. Fortunately, it was demonstrated that vancomycin resistance genes in lactobacilli are chromosomally encoded and cannot be transferred to other bacteria through conjugation [29]. Also, intrinsic vancomycin resistance in pediococci failed to transfer to enterococci through the filter matting method [30]. Despite reports of intrinsic aminoglycoside resistance among lactobacilli and pediococci [27, 31], genes encoding for aminoglycosides modifying enzymes, such as *aph*(*3*′)*-III* and *aac*(6′)-*aph*(2″), have been detected in *Lactobacillus* spp. as well as *Pediococcus* spp. [31, 32]. Unfortunately, such resistance determinants are generally reported to be localized on mobile genetic elements which pose an unequivocal threat to their potential transfer among bacterial species [26]. Genes encoding for resistance to β-lactams, such as *bla*Z, were previously reported to occur less frequently among *Lactobacillus* spp. [33]. In contrast, it was detected among the three tested LAB strains. Similarly, Aquilanti et al. detected *bla*Z gene among *L. plantarum* strains [34]. Despite the deficiency of reports of β-lactams resistance transferability in lactobacilli [35], transferrable resistance genes (*bla* and *bla*Z) can probably be transmitted through horizontal gene transfer when located on plasmids or transposons [36]. Acquired resistance to erythromycin among various *Lactobacillus* spp. including *L. plantarum* and *L. rhamnosus* has been reported to be due to *erm*B gene [25, 37, 38]. Moreover, the presence of such a gene was reported in *P. acidilactici* strains [31]. Investigations regarding the transferability of *erm*B gene are somehow contradictory. The lack of *erm*B transferability in *Lactobacillus* spp. has been detected [35]. On the contrary, many authors reported the localization of this gene on mobile genetic elements in *Lactobacillus* spp. or *Pediococcus* spp. This may lead to its conjugative transfer and thus raise concerns about the dissemination of acquired erythromycin resistance among LAB and other bacteria [28, 39].

A tested strain that is phenotypically resistant might be genotypically sensitive and, on the contrary, a susceptible phenotype may harbor silent genes which can be detected by molecular methods such as PCR [40]. In our study, the phenotypic-genotypic discrepancy in the resistance towards both ampicillin and erythromycin was observed among the tested strains. Such a lack of phenotypic-genotypic correlation has been reported in previous studies [27, 28, 34, 41].

Surprisingly, antibiotic resistance to multiple classes of antibiotics was prevalent in the tested LAB isolates obtained from marketed dietary supplements: P3, P4, and P5. Wong et al. have also reported the detection of antibiotic resistance to various broad-spectrum antibiotics in probiotic bacteria isolated from dietary supplements [10]. Such findings shed light on the importance of expanded phenotypic as well as genotypic screening for antibiotic resistance in probiotic strains incorporated in marketed products. Such antibiotic-resistant probiotic strains might act as reservoir organisms for antibiotic resistance genes that may pose an inevitable threat to the hosts.

Owing to their low potential for horizontal gene transfer, the presence of genes encoding for intrinsic mechanisms of antibiotic resistance in LAB is generally reported as an acceptable feature while selecting probiotics [42]. Despite previous reports concerning the absence of transferability of genes encoding antibiotic resistance from LAB strains to pathogenic bacterial strains [21], there is still an impending risk of transmission of acquired resistance genes among LAB and other bacterial species including pathogens. Consequently, the world of microbial biotherapy is directed towards the consumption of probiotic-derived components “postbiotics”, like the CFS of probiotics, as a safer alternative to the use of the whole viable probiotic microorganisms [6].

Notably, the probiotics’ non-neutralized CFS possessed remarkably strong inhibitory activity against the standard strains. However, after pH neutralization, the antimicrobial activity was markedly diminished. In concordance with our findings, it was reported that the antibacterial activity of the neutralized CFS of *Lactobacillus* strains was abolished compared to the non-neutralized CFS [15, 43]. Shukla et al. also detected the loss of inhibitory effect of the tested CFS of a LAB strain that belonged to *Pediococcus* spp. after pH neutralization [44]. The undissociated form of organic acids produced by probiotics contributes to the antimicrobial effect as it penetrates the bacterial cell membrane and releases hydrogen ions in the cytoplasm’s neutral environment. This results in a reduction in the intracellular pH which eventually hinders vital cell functions [45]. Moreover, it was reported that by lowering the pH value, the antimicrobial activity of bacteriocins is elevated sharply. At low pH, the secretion of hydrophobic bacteriocins is boosted so they can easily pass through the hydrophobic partitions of the cell wall. Also, at high pH, the binding of some bacteriocins to the cytoplasmic membrane’s receptor sites can be hindered [46]. Ohenhen et al. demonstrated that the bacteriocin extracted from *L. plantarum* strain showed a marked antimicrobial activity at pH 2 relative to that displayed at higher pH values. At pH 10, the antagonistic activity was completely eradicated [47].

Interestingly, in our study, there were obvious discrepancies regarding the antifungal activity of the tested LAB strains against *C. albicans* when assessed by two different methods: the agar overlay method and the microplate-based liquid medium assay. No antifungal activity was noticed by the agar overlay method. However, a marked inhibitory activity of the CFS was observed using the microplate-based liquid medium assay. Similarly, Wang et al. found that 100% of the examined CFS of the *Lactobacillus* strains showed anti-candida activities by the liquid medium assay, while only 83.3% of the tested strains exhibited antifungal activity against *C. albicans* in the agar overlay assay. Hence, they concluded that the liquid medium assay was likely to be more sensitive to evaluate the antagonistic activities compared with the solid agar-based assay. They attributed such variation to the fact that the solid agar might hinder the diffusion of inhibitory compounds to reach the target *C. albicans* [48].

Recently, probiotics are thought to be a promising tool for combating infectious biofilms through various mechanisms. These mechanisms include the production of many metabolites (e.g., organic acids, exopolysaccharides, bacteriocins, and biosurfactants) with antibiofilm activity. Also, probiotics can participate in the creation of undesirable environmental conditions for the pathogens that might hinder their survival mainly through alteration of pH and competition for nutrients and surface. Additionally, probiotics can influence the expression of genes contributing to the production of pathogenic biofilms and can also impede quorum sensing systems [49]. *P. aeruginosa* and *S. aureus* are considered among the most challenging biofilm formers that can cause life-threatening infections [49, 50]. In our study, the nCFS of the tested probiotics could inhibit the biofilm formation of the tested *S. aureus* isolates as well as *P. aeruginosa* strains but to different extents. In agreement with our results, the nCFS of *Lactobacillus* strains, including *L. plantarum*, as well as *P. acidilactici* could inhibit the biofilm formation of *S. aureus* [51]. Other studies reported the ability of *Lactobacillus* spp. and *Pediococcus* spp. probiotic strains to inhibit the biofilm formation in *P. aeruginosa* [52, 53].

Carrageenan-induced acute edema in Wistar rats is thought to be a suitable model for the assessment of anti-inflammatory agents. The inflammatory response to carrageenan is biphasic in nature. The initial phase a few hours post injection is attributed to the release of histamine, serotonin, and kinins. In the second phase, the release of prostaglandins, the principal mediators of acute inflammation, is evident [54]. Indomethacin has been previously used as a standard anti-inflammatory agent in the carrageenan-induced rat paw edema model [55, 56]. Indomethacin, a potent non-steroidal anti-inflammatory drug, possesses a wide array of applications owing to its anti-inflammatory, antipyretic, and analgesic activities. The main mechanism underlying such activities is the inhibition of the synthesis of prostaglandins that are primarily produced by cyclooxygenase enzymes which are considered to be essential mediators of inflammation, pain, and fever [57].

The present study showed that the cell cultures of the tested *Lactobacillus* spp. were able to reduce the acute inflammation induced by carrageenan when tested in male Wistar rats. Similarly, Archer et al. and Ayyanna et al. demonstrated an anti-inflammatory activity of *Lactobacillus* strains by significantly reducing the rat paw inflammation and edema induced by carrageenan. It was hypothesized that *Lactobacillus* spp. strains could inhibit the cyclooxygenase pathway, which is important in prostaglandin synthesis, in addition to their ability to modulate cytokines secretion. This resulted in an overall amelioration of inflammation [54, 58]. Probiotics may also contribute to the upregulation of the powerful immunomodulators, regulatory T-cells (T-regs), which can lead to a significant overall downregulation of the pro-inflammatory cascade of reactions [59].

To avoid the potential risks associated with the consumption of probiotics as whole viable cells, postbiotic supernatant could be used to achieve an immune modulation [59]. In our study, the anti-inflammatory effect of the studied CFS was not profoundly evident as that observed in the case of the probiotic cultures. However, the CFS of both P3 and P4 were able to relatively reduce the histological inflammatory changes resulting from carrageenan injection compared to the inflammation control group. To the best of our knowledge, the carrageenan-induced rat paw edema model in male Wistar rats was not previously experimented to determine the anti-inflammatory activity of the CFS of probiotics. However, other in vitro as well as a few in vivo models have proved the anti-inflammatory activity of probiotics’ CFS [60, 61]. In our study, the less obvious anti-inflammatory effect of CFS, compared to the probiotic cultures, could be attributed to the possible improper selection of the dose or the concentration of the metabolites to be administered to the rats. The CFS of the tested probiotics could be additionally tested in other in vivo models with further optimization of the experimental conditions. Besides, the protective effects of probiotics are thought to be strain-specific [62]. However, it is important to note that the current study has a potential limitation. It would have been useful if further studies like cytokine measurement of the postbiotic in the rat model by quantitative polymerase chain reaction or ELISA or characterization of the postbiotic could have been included for better investigation of the anti-inflammatory potential of the CFS of the tested probiotic strains.

## Conclusions

In conclusion, the tested probiotics, as well as their CFS, showed promising antimicrobial and anti-inflammatory activities. The present study paves the way for further future research work focusing on the investigation of the biotherapeutic potential of a larger number of probiotic strains from different genera, along with their supernatants, to be utilized against various bacterial infections and inflammatory conditions. Besides, their safety should be strictly monitored especially regarding the potential presence and transferability of acquired antibiotic resistance genes.

## Methods

### Isolation of probiotic strains from commercially available dietary supplements

The study included three commercially available probiotic dietary supplements, manufactured in the USA, and designated here as DSP3, DSP4, and DSP5 (Table 1). To recover probiotic bacteria, one capsule of each dietary supplement was aseptically inoculated into De Man, Rogosa, Sharpe (MRS) broth (Himedia, India) and incubated aerobically at 37 °C for 24–48 h. Pure cultures were obtained after streaking on MRS agar plates. Identification of the recovered probiotic strains was done using MALDI-TOF MS (Bruker Daltonik, USA).

### Evaluation of the antimicrobial activity of probiotic strains

The antimicrobial activity of the probiotic strains against *Escherichia coli* NCTC 10418, *Staphylococcus aureus* ATCC 6538, *Salmonella enterica subsp. enterica serovar* Typhimurium ATCC 14028, *Listeria monocytogenes* EGD-e (serotype 1/2a), and *Candida albicans* ATCC 10231 was determined using the agar overlay technique. Two microliters of the overnight culture of each probiotic strain were inoculated as a single spot on the surface of MRS agar plates. Then, the plates were allowed to dry at room temperature for 30 min before aerobic incubation for 24–48 h at 37 °C. After colony development, the plates were overlaid with a volume of 10 mL of soft (0.6% (w/v) agar agar) Müller-Hinton medium (Lab M, UK), or Sabouraud dextrose medium (Oxoid, England), for *C. albicans*, seeded with an overnight culture of the indicator strains to reach a final count of 10^6^ CFU/mL. A hundred microliters and 1 mL of bacteria and *Candida* cultures, respectively, were inoculated into 10 mL of soft agar to reach the required organism’s final count. The plates were then aerobically incubated for 24–48 h at 37 °C. The inhibition zones developed around the spots of probiotic strains were measured and the results were interpreted as follows: zones of more than 20 mm indicated strong inhibition activity, zones of 10 to 20 mm designated intermediate inhibition potential, and zones less than 10 mm were indicative of low inhibition activity [43].

### Phenotypic characterization of selected virulence factors among the tested probiotic strains

To investigate the hemolytic activity of the tested probiotic strains, fresh cultures were streaked on Columbia agar plates (Himedia, India), containing 5% (w/v) human blood, then incubated at 37 °C for 48 h to be examined for α, β, and γ -hemolysis [63]. For the detection of the gelatinase activity, 10 µL of the overnight cultures were spot inoculated on sterile gelatin agar plates, incubated for 48 h at 37 °C, and then flooded with 10 mL of saturated ammonium sulfate. The formation of clear zones around the colonies was indicative of gelatinase activity. To screen for the DNase enzyme activity, overnight cultures of the tested strains were 10 µL spot inoculated on sterile DNase agar plates (Lab M, UK). After incubation at 37 °C for 48 h, the plates were flooded with 10 mL of 1 N HCl. The formation of clear zones around the colonies indicated DNase production [64]. *S. aureus* ATCC 6538 was included as a positive control in all the virulence tests.

### Antimicrobial susceptibility testing

The susceptibility of the tested probiotic strains to 5 antibiotics: ampicillin, kanamycin, gentamicin, vancomycin, and erythromycin was determined using the broth microdilution technique. The overnight culture of each strain was centrifuged at 7000 rpm for 10 min at 4 °C and cell pellets were resuspended in saline and adjusted to OD_600_ nm ca. 0.2. The culture was then 100-fold diluted in double strength (D/S) MRS broth. A sterile 96-well microtiter plate was inoculated with 100 µL of diluted inoculum and 100 µL of 2-fold serially diluted antibiotics solutions to reach a final bacterial inoculum of about 5 × 10^5^ CFU/mL. The sterilized medium control and growth control wells were also included in the experiment. After aerobic incubation for 48 h at 37 °C, the minimum inhibitory concentrations (MICs) were calculated based on the absorbance readings (OD_630_ nm) obtained using a microtiter plate reader [15, 65], and the results were interpreted based on the European Food Safety Authority (EFSA) guidelines (2018) [66].

### Detection of antibiotic resistance genes using polymerase chain reaction (PCR)

DNA extraction from probiotic strains was carried out as formerly described [67]. The amplification of selected antibiotic resistance genes ((*aac(6’)-aph(2”)* [68], *aph(3’’)-III* [68], *van*X [69], *erm*B [68], *bla*Z [41], and *bla* [69]) was done using PCR. The primers used are mentioned in Additional file 1 and the applied thermal cycling conditions are illustrated in Additional file 2. PCR products were detected using 1% agarose gel electrophoresis in Tris Acetate EDTA (TAE) buffer. The bands of the PCR products were visualized under a UV transilluminator (High-Performance UV transilluminator, USA) at 254 nm. Sizes of the obtained bands were determined corresponding to the loaded 100 bp DNA ladder (Thermo Fisher Scientific, UK).

### Assessment of the mechanism of the antimicrobial activity of the tested probiotic strains

The antimicrobial activity of the cell-free supernatant (CFS) of the probiotic strains was determined, using the microtiter plate assay, against the formerly stated standard strains. For the preparation of CFS of probiotic strains, each strain was propagated in MRS broth at 37 °C for 48 h, centrifuged, and then the supernatant was filtered using a syringe filter (0.22 μm pore size) (Filter-bio Co., China). One portion of the prepared CFS maintained its initial acidic pH while the second one (nCFS) was neutralized using 5 M NaOH to reach pH 6.5. A sterile 96-well microtiter plate was loaded with 100 µL of CFS (or nCFS) and 100 µL of D/S Luria Bertani (LB) broth, or D/S Sabouraud dextrose broth for *C. albicans*, with the inoculated organism (a final count of 10^6^ CFU per well). The sterilized medium control and growth control wells were also included in the experiment. After incubating the plates at 37 °C for 24 h, the optical density (OD) was measured at 630 nm. The total percentage inhibition of bacterial growth was determined as: Percentage inhibition = [(OD of the positive control - OD of the test sample) / OD of the positive control] x 100 [70].

### Evaluation of the effect of nCFS on the biofilm formation of selected pathogenic strains

The anti-biofilm activity of the nCFS of the probiotic strains was tested against representative challenging biofilm former clinical isolates: two *S. aureus* clinical isolates isolated from urinary tract infection (UTI) (*S. aureus*^UTI1^ and *S. aureus*^UTI2^) and one *Pseudomonas aeruginosa* clinical isolate from pus (*P. aeruginosa*^pus^). In addition, the standard biofilm former strain *P. aeruginosa* PAO1 strain was included in the experiment. CFS of the tested probiotic strains were prepared as previously mentioned, utilizing Tween 80- free MRS broth [71], then neutralized to pH 6.5 using 5 M NaOH. Each pathogenic bacterial strain was overnight cultured in sterile tryptone soya broth supplemented with glucose (0.5% (w/v)). Hundred microliters of the diluted culture of each pathogen were transferred to a 96-well microtiter plate and 100 µL of each probiotic nCFS were added such that the final count of each pathogenic culture was ca. 10^6^ CFU/mL. After overnight incubation at 37 °C, the medium was rejected, and the plates were gently washed twice using sterile PBS to remove the planktonic cells from each well. Biofilms were then fixed with 200 µL methanol for 15 min, stained for 20 min with 200 µL of crystal violet (1%), and then gently washed thrice with water. Dissolving crystal violet dye attached to the biofilm samples was done using 200 µL of glacial acetic acid (33%). After measuring the absorbance at 630 nm, the percentage of biofilm inhibition was determined as: Percentage of biofilm inhibition = 100 – [(OD_630_ of wells in the presence of probiotic CFS x 100)/ OD_630_ of wells in the presence of MRS broth] [51, 72].

### Assessment of the anti-inflammatory activity of *Lactobacillus* spp. probiotic strains on carrageenan-induced paw edema model in male Wistar rats

#### Animals

Forty-two male adult Wistar rats acquired from the animal facility of the National Institute of Oncology, Cairo, Egypt, with an average weight of 180–220 g (10–13 weeks old) were included in this study. During the acclimatization period, the animals were housed under controlled laboratory conditions ensuring free access to an ad libitum supply of standard rodent chow and water.

#### Preparation of probiotics cell cultures and CFS

*Lactiplantibacillus plantarum* P3 and *Lacticaseibacillus rhamnosus* P4, isolated from probiotic dietary supplements DSP3 and DSP4, respectively, were inoculated in 20 mL sterile MRS broth and incubated for 24 h at 37 °C. For the preparation of the CFS, each strain was subcultured in 5 falcons each containing 30 mL sterile MRS broth for 48 h at 37 °C. Then, to obtain the CFS, the culture was centrifuged and supernatants from each of the five falcons were pooled in a sterile container to obtain 150 mL total supernatant volume which was then filtered through a 0.22 μm pore size syringe filter and stored in the − 20 °C freezer.

#### Experimental protocol

The experiment lasted for 8 days [54]. The Wistar rats were grouped as follows (n = 6 per group): (i) Group A (saline control), (ii) Group B (carrageenan control), (iii) Group C (indomethacin group, a standard anti-inflammatory model), (iv) Group D received 1 mL of *L. rhamnosus* culture 10^8^ CFU/mL/day [58], (v) Group E received 2 mL of *L. rhamnosus* CFS/day, (vi) Group F received 1 mL of *L. plantarum* culture 10^8^ CFU/mL/day, and (vii) Group G received 2 mL of *L. plantarum* CFS/day.

Groups D to G were administered their respective regimens orally for 8 days. Group C received 1 mL of indomethacin (10 mg/kg) on the 8th day [73].

#### Induction of rat paw edema using carrageenan

On the 8th day, and 1 h after oral treatments [55], 100 µL of fresh carrageenan (Sigma-Aldrich Co., USA) solution (1%) were subplantar injected into the right hind paw of each rat except for the rats of the control group A that were injected with 100 µL saline [58]. To gauge the extent of inflammation, the paw thickness of the rats was measured just before the carrageenan injection as well as at 1, 2, 3, 4, and 5 h after the carrageenan injection using a manual Vernier caliper [74]. The percentage increase in the paw thickness was subsequently calculated at each time interval [75]. After the final paw measurement at 5 h, the rats were euthanized by an overdose of a general anesthetic (thiopental sodium, 50 mg/kg), and the right hind paws were harvested for histopathological studies.

#### Histopathological evaluation of inflammatory changes

The inflammatory changes elicited in tissues following the subplantar injection of carrageenan/saline into the rats’ right hind paw were assessed by histopathological evaluation of the formalin fixed paraffin embedded paw tissue sections from the 6 rats in each group using the hematoxylin and eosin (H&E) stain [58].

A semiquantitative scoring was done according to Coura et al. [76] with some modifications. Separate scoring of each inflammatory feature like congestion, edema, necrosis as well as the intensity of inflammatory infiltrate in the rat paw tissues was performed using a scale from 0 to 3 (0, + 1, +2, + 3) where 0 = not present, + 1 = mild, + 2 = moderate, and + 3 = severe. Then, a total histologic score (out of 12) was calculated by adding together the scores for each inflammatory feature.

#### Statistical analysis

All values were expressed as means ± standard error of the mean (SEM). The one or two-way analysis of variance (ANOVA) followed by Tukey’s post hoc test was employed to determine the statistical significance where the level of significance was set at p < 0.05. All analyses were done utilizing GraphPad Prism software, version. 6.01.

## Electronic supplementary material

Below is the link to the electronic supplementary material.

**Additional file 1:** Oligonucleotide primers used for the amplification of antibiotic resistance genes.

**Additional file 2:** PCR amplification conditions of the antibiotic resistance genes.

**Additional file 3:** Statistical analysis of the percentage increase in the paw thickness of Wistar rats of the control and treated groups with whole cell culture and CFS of *L. rhamnosus* (P4).

**Additional file 4:** Statistical analysis of the percentage increase in the paw thickness of Wistar rats of the control and treated groups with whole cell culture and CFS of *L. plantarum* (P3).

## Data Availability

Most data generated or analyzed during this study are included in this published article and its additional files. Any extra demanded details are available from the corresponding author on reasonable request.

## List of abbreviations

CFS: Cell-free supernatants
LAB: Lactic acid bacteria
MRS: De Man, Rogosa, Sharpe
MALDI-TOF MS: Matrix-assisted laser desorption ionization time-of-flight mass spectrometry
ATCC: American Type Culture Collection
NCTC: National Collection of Type Cultures
DNase: Deoxyribonuclease
MIC: Minimum inhibitory concentration
EFSA: European Food Safety Authority
PCR: Polymerase chain reaction
TAE: Tris Acetate EDTA
LB: Luria Bertani
nCFS: Neutralized CFS
H&E: Hematoxylin and eosin
ANOVA: Analysis of variance
T-regs: Regulatory T-cells
