# Supplementary material for: Promising biotherapeutic prospects of different probiotics and their derived postbiotic metabolites: in-vitro and histopathological investigation
Source: BMC Microbiol. 2023 May 3;23:122. doi: 10.1186/s12866-023-02866-1 (PMC10155454; doi:10.1186/s12866-023-02866-1)
Supplement: Supplementary file 1 — Additional file 1: Oligonucleotide primers used for the amplification of antibiotic resistance genes. [file 12866_2023_2866_MOESM1_ESM.docx]

**Additional file 1:** Oligonucleotide primers used for the amplification of antibiotic resistance genes.

| Target gene | Primer name |  | Nucleotide sequence (5′→3′) | Melting temperature  (Tm °C) | Size of the  amplicons (bps) |
| --- | --- | --- | --- | --- | --- |
| *ermB* | *erm*B-F |  | CATTTAACGACGAAACTGGC | 52.2 | 425 |
|  | *erm*B-R |  | GGAACATCTGTGGTATGGCG | 55.9 |  |
| *aac(6’)-aph(2’’)* | *aac(6’)- aph(2’’)*-F |  | CCAAGAGCAATAAGGGCATA | 52.3 | 220 |
|  | *aac(6’)- aph(2’’)*-R |  | CACTATCATAACCACTACCG | 49.7 |  |
| *aph(3’’)-III* | *aph(3’’)-III*-F |  | GCCGATGTGGATTGCGAAAA | 56.6 | 292 |
|  | *aph(3’’)-III*-R |  | GCTTGATCCCCAGTAAGTCA | 54.3 |  |
| *bla* | *bla*-F |  | CATARTTCCGATAATASMGCC | 50.6 | 297 |
|  | *bla*-R |  | CGTSTTTAACTAAGTATSGY | 47.7 |  |
| *blaZ* | *blaZ*-F |  | ACTTCAACACCTGCTGCTTTC | 55.9 | 240 |
|  | *blaZ*-R |  | TAGGTTCAGATTGGCCCTTAG | 53.9 |  |
| *van*X | *van*X-F |  | TCGCGGTAGTCCCACCATTCGTT | 62.6 | 454 |
|  | *van*X-R |  | AAATCATCGTTGACCTGCGTTAT | 55 |  |
