# Supplementary material for: Promising biotherapeutic prospects of different probiotics and their derived postbiotic metabolites: in-vitro and histopathological investigation
Source: BMC Microbiol. 2023 May 3;23:122. doi: 10.1186/s12866-023-02866-1 (PMC10155454; doi:10.1186/s12866-023-02866-1)
Supplement: Supplementary file 2 — Additional file 2: PCR amplification conditions of the antibiotic resistance genes. [file 12866_2023_2866_MOESM2_ESM.docx]

**Additional file 2:** PCR amplification conditions of the antibiotic resistance genes.

| Antibiotic resistance gene | Thermal cycling conditions | | | | |
| --- | --- | --- | --- | --- | --- |
|  | **Initial denaturation** | **35 cycles** | | | **Final extension** |
|  |  | **Denaturation** | **Annealing** | **Extension** |  |
| *erm*B | 95 °C/1 min | 95 °C/15 s | 48 °C/15 s | 72 °C/20 s | 72 °C/10 mins |
| *aac(6’)-aph(2’’)* |  |  | 51 °C/15 s |  |  |
| *aph(3’’)-III* |  |  | 51 °C/15 s |  |  |
| *bla* |  |  | 46 °C/15 s |  |  |
| *blaZ* |  |  | 51 °C/15 s |  |  |
| *van*X |  |  | 51 °C/15 s |  |  |
