# Supplementary material for: Promising biotherapeutic prospects of different probiotics and their derived postbiotic metabolites: in-vitro and histopathological investigation
Source: BMC Microbiol. 2023 May 3;23:122. doi: 10.1186/s12866-023-02866-1 (PMC10155454; doi:10.1186/s12866-023-02866-1)
Supplement: Supplementary file 3 — Additional file 3: Statistical analysis of the percentage increase in the paw thickness of Wistar rats of the control and treated groups with whole cell culture and CFS of L. rhamnosus (P4). [file 12866_2023_2866_MOESM3_ESM.docx]

**Additional file 3:** Statistical analysis of the percentage increase in the paw thickness of Wistar rats of the control and treated groups with whole cell culture and CFS of *L. rhamnosus* (P4).

| **Tukey's multiple comparisons test** | **Mean difference** | **95% CI^a^ of difference** | **Significance** | **Adjusted P- value** |
| --- | --- | --- | --- | --- |
|  |  |  |  |  |
| **0 hr** | | | | |
| Saline vs. Carrageenan | 0.0 | -15.13 to 15.13 | NS^b^ | > 0.9999 |
| Saline vs. Indomethacin / Carrageenan | 0.0 | -15.13 to 15.13 | NS | > 0.9999 |
| Saline vs. Whole cell culture (P4) / Carrageenan | 0.0 | -15.13 to 15.13 | NS | > 0.9999 |
| Saline vs. CFS (P4) / Carrageenan | 0.0 | -15.13 to 15.13 | NS | > 0.9999 |
| Carrageenan vs. Indomethacin / Carrageenan | 0.0 | -15.13 to 15.13 | NS | > 0.9999 |
| Carrageenan vs. Whole cell culture (P4) / Carrageenan | 0.0 | -15.13 to 15.13 | NS | > 0.9999 |
| Carrageenan vs. CFS (P4) / Carrageenan | 0.0 | -15.13 to 15.13 | NS | > 0.9999 |
| Indomethacin / Carrageenan vs. Whole cell culture (P4) / Carrageenan | 0.0 | -15.13 to 15.13 | NS | > 0.9999 |
| Indomethacin / Carrageenan vs. CFS (P4) / Carrageenan | 0.0 | -15.13 to 15.13 | NS | > 0.9999 |
| Whole cell culture (P4) / Carrageenan vs. CFS (P4) / Carrageenan | 0.0 | -15.13 to 15.13 | NS | > 0.9999 |
|  |  |  |  |  |
| **1 hr** | | | | |
| Saline vs. Carrageenan | -31.61 | -46.74 to -16.48 | **** | < 0.0001 |
| Saline vs. Indomethacin / Carrageenan | -8.170 | -23.30 to 6.956 | NS | 0.5697 |
| Saline vs. Whole cell culture (P4) / Carrageenan | -14.87 | -30.00 to 0.2562 | NS | 0.0565 |
| Saline vs. CFS (P4) / Carrageenan | -16.59 | -31.72 to -1.464 | * | 0.0238 |
| Carrageenan vs. Indomethacin / Carrageenan | 23.44 | 8.314 to 38.57 | *** | 0.0003 |
| Carrageenan vs. Whole cell culture (P4) / Carrageenan | 16.74 | 1.614 to 31.87 | * | 0.0220 |
| Carrageenan vs. CFS (P4) / Carrageenan | 15.02 | -0.1062 to 30.15 | NS | 0.0526 |
| Indomethacin / Carrageenan vs. Whole cell culture (P4) / Carrageenan | -6.700 | -21.83 to 8.426 | NS | 0.7380 |
| Indomethacin / Carrageenan vs. CFS (P4) / Carrageenan | -8.420 | -23.55 to 6.706 | NS | 0.5402 |
| Whole cell culture (P4) / Carrageenan vs. CFS (P4) / Carrageenan | -1.720 | -16.85 to 13.41 | NS | 0.9979 |
|  |  |  |  |  |
| **2 hrs** | | | | |
| Saline vs. Carrageenan | -44.71 | -59.84 to -29.58 | **** | < 0.0001 |
| Saline vs. Indomethacin / Carrageenan | -8.610 | -23.74 to 6.516 | NS | 0.5178 |
| Saline vs. Whole cell culture (P4) / Carrageenan | -28.34 | -43.47 to -13.21 | **** | < 0.0001 |
| Saline vs. CFS (P4) / Carrageenan | -28.96 | -44.09 to -13.83 | **** | < 0.0001 |
| Carrageenan vs. Indomethacin / Carrageenan | 36.10 | 20.97 to 51.23 | **** | < 0.0001 |
| Carrageenan vs. Whole cell culture (P4) / Carrageenan | 16.37 | 1.244 to 31.50 | * | 0.0268 |
| Carrageenan vs. CFS (P4) / Carrageenan | 15.75 | 0.6238 to 30.88 | * | 0.0368 |
| Indomethacin / Carrageenan vs. Whole cell culture (P4) / Carrageenan | -19.73 | -34.86 to -4.604 | ** | 0.0039 |
| Indomethacin / Carrageenan vs. CFS (P4) / Carrageenan | -20.35 | -35.48 to -5.224 | ** | 0.0026 |
| Whole cell culture (P4) / Carrageenan vs. CFS (P4) / Carrageenan | -0.6200 | -15.75 to 14.51 | NS | > 0.9999 |
|  |  |  |  |  |
| **3 hrs** | | | | |
| Saline vs. Carrageenan | -53.78 | -68.91 to -38.65 | **** | < 0.0001 |
| Saline vs. Indomethacin / Carrageenan | -11.31 | -26.44 to 3.816 | NS | 0.2410 |
| Saline vs. Whole cell culture (P4) / Carrageenan | -30.86 | -45.99 to -15.73 | **** | < 0.0001 |
| Saline vs. CFS (P4) / Carrageenan | -33.46 | -48.59 to -18.33 | **** | < 0.0001 |
| Carrageenan vs. Indomethacin / Carrageenan | 42.47 | 27.34 to 57.60 | **** | < 0.0001 |
| Carrageenan vs. Whole cell culture (P4) / Carrageenan | 22.92 | 7.794 to 38.05 | *** | 0.0005 |
| Carrageenan vs. CFS (P4) / Carrageenan | 20.32 | 5.194 to 35.45 | ** | 0.0027 |
| Indomethacin / Carrageenan vs. Whole cell culture (P4) / Carrageenan | -19.55 | -34.68 to -4.424 | ** | 0.0043 |
| Indomethacin / Carrageenan vs. CFS (P4) / Carrageenan | -22.15 | -37.28 to -7.024 | *** | 0.0008 |
| Whole cell culture (P4) / Carrageenan vs. CFS (P4) / Carrageenan | -2.600 | -17.73 to 12.53 | NS | 0.9895 |
|  |  |  |  |  |
| **4 hrs** | | | | |
| Saline vs. Carrageenan | -50.37 | -65.50 to -35.24 | **** | < 0.0001 |
| Saline vs. Indomethacin / Carrageenan | -11.35 | -26.48 to 3.776 | NS | 0.2378 |
| Saline vs. Whole cell culture (P4) / Carrageenan | -31.42 | -46.55 to -16.29 | **** | < 0.0001 |
| Saline vs. CFS (P4) / Carrageenan | -37.86 | -52.99 to -22.73 | **** | < 0.0001 |
| Carrageenan vs. Indomethacin / Carrageenan | 39.02 | 23.89 to 54.15 | **** | < 0.0001 |
| Carrageenan vs. Whole cell culture (P4) / Carrageenan | 18.95 | 3.824 to 34.08 | ** | 0.0063 |
| Carrageenan vs. CFS (P4) / Carrageenan | 12.51 | -2.616 to 27.64 | NS | 0.1559 |
| Indomethacin / Carrageenan vs. Whole cell culture (P4) / Carrageenan | -20.07 | -35.20 to -4.944 | ** | 0.0031 |
| Indomethacin / Carrageenan vs. CFS (P4) / Carrageenan | -26.51 | -41.64 to -11.38 | **** | < 0.0001 |
| Whole cell culture (P4) / Carrageenan vs. CFS (P4) / Carrageenan | -6.440 | -21.57 to 8.686 | NS | 0.7653 |
|  |  |  |  |  |
| **5 hrs** | | | | |
| Saline vs. Carrageenan | -53.88 | -69.01 to -38.75 | **** | < 0.0001 |
| Saline vs. Indomethacin / Carrageenan | -13.22 | -28.35 to 1.906 | NS | 0.1174 |
| Saline vs. Whole cell culture (P4) / Carrageenan | -34.74 | -49.87 to -19.61 | **** | < 0.0001 |
| Saline vs. CFS (P4) / Carrageenan | -40.85 | -55.98 to -25.72 | **** | < 0.0001 |
| Carrageenan vs. Indomethacin / Carrageenan | 40.66 | 25.53 to 55.79 | **** | < 0.0001 |
| Carrageenan vs. Whole cell culture (P4) / Carrageenan | 19.14 | 4.014 to 34.27 | ** | 0.0056 |
| Carrageenan vs. CFS (P4) / Carrageenan | 13.03 | -2.096 to 28.16 | NS | 0.1269 |
| Indomethacin / Carrageenan vs. Whole cell culture (P4) / Carrageenan | -21.52 | -36.65 to -6.394 | ** | 0.0012 |
| Indomethacin / Carrageenan vs. CFS (P4) / Carrageenan | -27.63 | -42.76 to -12.50 | **** | < 0.0001 |
| Whole cell culture (P4) / Carrageenan vs. CFS (P4) / Carrageenan | -6.110 | -21.24 to 9.016 | NS | 0.7982 |

^a^CI: Confidence interval, ^b^NS: Non-significant, * < 0.05, ** < 0.01, *** P < 0.001, **** P < 0.0001.
