# Supplementary material for: Promising biotherapeutic prospects of different probiotics and their derived postbiotic metabolites: in-vitro and histopathological investigation
Source: BMC Microbiol. 2023 May 3;23:122. doi: 10.1186/s12866-023-02866-1 (PMC10155454; doi:10.1186/s12866-023-02866-1)
Supplement: Supplementary file 4 — Additional file 4: Statistical analysis of the percentage increase in the paw thickness of Wistar rats of the control and treated groups with whole cell culture and CFS of L. plantarum (P3). [file 12866_2023_2866_MOESM4_ESM.docx]

**Additional file 4:** Statistical analysis of the percentage increase in the paw thickness of Wistar rats of the control and treated groups with whole cell culture and CFS of *L. plantarum* (P3).

| **Tukey's multiple comparisons test** | **Mean difference** | **95% CI^a^ of difference** | **Significance** | **Adjusted P- Value** |
| --- | --- | --- | --- | --- |
|  |  |  |  |  |
| **0 hr** | | | | |
| Saline vs. Carrageenan | 0.0 | -12.52 to 12.52 | NS^b^ | > 0.9999 |
| Saline vs. Indomethacin / Carrageenan | 0.0 | -12.52 to 12.52 | NS | > 0.9999 |
| Saline vs. Whole cell culture (P3) / Carrageenan | 0.0 | -12.52 to 12.52 | NS | > 0.9999 |
| Saline vs. CFS (P3) / Carrageenan | 0.0 | -12.52 to 12.52 | NS | > 0.9999 |
| Carrageenan vs. Indomethacin / Carrageenan | 0.0 | -12.52 to 12.52 | NS | > 0.9999 |
| Carrageenan vs. Whole cell culture (P3) / Carrageenan | 0.0 | -12.52 to 12.52 | NS | > 0.9999 |
| Carrageenan vs. CFS (P3) / Carrageenan | 0.0 | -12.52 to 12.52 | NS | > 0.9999 |
| Indomethacin / Carrageenan vs. Whole cell culture (P3) / Carrageenan | 0.0 | -12.52 to 12.52 | NS | > 0.9999 |
| Indomethacin / Carrageenan vs. CFS (P3) / Carrageenan | 0.0 | -12.52 to 12.52 | NS | > 0.9999 |
| Whole cell culture (P3) / Carrageenan vs. CFS (P3) / Carrageenan | 0.0 | -12.52 to 12.52 | NS | > 0.9999 |
|  |  |  |  |  |
| **1 hr** | | | | |
| Saline vs. Carrageenan | -31.61 | -44.13 to -19.09 | **** | < 0.0001 |
| Saline vs. Indomethacin / Carrageenan | -8.170 | -20.69 to 4.351 | NS | 0.3765 |
| Saline vs. Whole cell culture (P3) / Carrageenan | -13.25 | -25.77 to -0.7286 | * | 0.0323 |
| Saline vs. CFS (P3) / Carrageenan | -19.02 | -31.54 to -6.499 | *** | 0.0004 |
| Carrageenan vs. Indomethacin / Carrageenan | 23.44 | 10.92 to 35.96 | **** | < 0.0001 |
| Carrageenan vs. Whole cell culture (P3) / Carrageenan | 18.36 | 5.839 to 30.88 | *** | 0.0008 |
| Carrageenan vs. CFS (P3) / Carrageenan | 12.59 | 0.06860 to 25.11 | * | 0.0480 |
| Indomethacin / Carrageenan vs. Whole cell culture (P3) / Carrageenan | -5.080 | -17.60 to 7.441 | NS | 0.7956 |
| Indomethacin / Carrageenan vs. CFS (P3) / Carrageenan | -10.85 | -23.37 to 1.671 | NS | 0.1230 |
| Whole cell culture (P3) / Carrageenan vs. CFS (P3) / Carrageenan | -5.770 | -18.29 to 6.751 | NS | 0.7085 |
|  |  |  |  |  |
| **2 hrs** | | | | |
| Saline vs. Carrageenan | -44.71 | -57.23 to -32.19 | **** | < 0.0001 |
| Saline vs. Indomethacin / Carrageenan | -8.610 | -21.13 to 3.911 | NS | 0.3225 |
| Saline vs. Whole cell culture (P3) / Carrageenan | -26.23 | -38.75 to -13.71 | **** | < 0.0001 |
| Saline vs. CFS (P3) / Carrageenan | -27.79 | -40.31 to -15.27 | **** | < 0.0001 |
| Carrageenan vs. Indomethacin / Carrageenan | 36.10 | 23.58 to 48.62 | **** | < 0.0001 |
| Carrageenan vs. Whole cell culture (P3) / Carrageenan | 18.48 | 5.959 to 31.00 | *** | 0.0007 |
| Carrageenan vs. CFS (P3) / Carrageenan | 16.92 | 4.399 to 29.44 | ** | 0.0025 |
| Indomethacin / Carrageenan vs. Whole cell culture (P3) / Carrageenan | -17.62 | -30.14 to -5.099 | ** | 0.0014 |
| Indomethacin / Carrageenan vs. CFS (P3) / Carrageenan | -19.18 | -31.70 to -6.659 | *** | 0.0004 |
| Whole cell culture (P3) / Carrageenan vs. CFS (P3) / Carrageenan | -1.560 | -14.08 to 10.96 | ns | 0.9970 |
| **3 hrs** | | | | |
| Saline vs. Carrageenan | -53.78 | -66.30 to -41.26 | **** | < 0.0001 |
| Saline vs. Indomethacin / Carrageenan | -11.31 | -23.83 to 1.211 | NS | 0.0974 |
| Saline vs. Whole cell culture (P3) / Carrageenan | -24.35 | -36.87 to -11.83 | **** | < 0.0001 |
| Saline vs. CFS (P3) / Carrageenan | -36.67 | -49.19 to -24.15 | **** | < 0.0001 |
| Carrageenan vs. Indomethacin / Carrageenan | 42.47 | 29.95 to 54.99 | **** | < 0.0001 |
| Carrageenan vs. Whole cell culture (P3) / Carrageenan | 29.43 | 16.91 to 41.95 | **** | < 0.0001 |
| Carrageenan vs. CFS (P3) / Carrageenan | 17.11 | 4.589 to 29.63 | ** | 0.0021 |
| Indomethacin / Carrageenan vs. Whole cell culture (P3) / Carrageenan | -13.04 | -25.56 to -0.5186 | * | 0.0367 |
| Indomethacin / Carrageenan vs. CFS (P3) / Carrageenan | -25.36 | -37.88 to -12.84 | **** | < 0.0001 |
| Whole cell culture (P3) / Carrageenan vs. CFS (P3) / Carrageenan | -12.32 | -24.84 to 0.2014 | NS | 0.0562 |
|  |  |  |  |  |
| **4 hrs** | | | | |
| Saline vs. Carrageenan | -50.37 | -62.89 to -37.85 | **** | < 0.0001 |
| Saline vs. Indomethacin / Carrageenan | -11.35 | -23.87 to 1.171 | NS | 0.0954 |
| Saline vs. Whole cell culture (P3) / Carrageenan | -26.15 | -38.67 to -13.63 | **** | < 0.0001 |
| Saline vs. CFS (P3) / Carrageenan | -34.70 | -47.22 to -22.18 | **** | < 0.0001 |
| Carrageenan vs. Indomethacin / Carrageenan | 39.02 | 26.50 to 51.54 | **** | < 0.0001 |
| Carrageenan vs. Whole cell culture (P3) / Carrageenan | 24.22 | 11.70 to 36.74 | **** | < 0.0001 |
| Carrageenan vs. CFS (P3) / Carrageenan | 15.67 | 3.149 to 28.19 | ** | 0.0063 |
| Indomethacin / Carrageenan vs. Whole cell culture (P3) / Carrageenan | -14.80 | -27.32 to -2.279 | * | 0.0117 |
| Indomethacin / Carrageenan vs. CFS (P3) / Carrageenan | -23.35 | -35.87 to -10.83 | **** | < 0.0001 |
| Whole cell culture (P3) / Carrageenan vs. CFS (P3) / Carrageenan | -8.550 | -21.07 to 3.971 | NS | 0.3296 |
|  |  |  |  |  |
| **5 hrs** | | | | |
| Saline vs. Carrageenan | -53.88 | -66.40 to -41.36 | **** | < 0.0001 |
| Saline vs. Indomethacin / Carrageenan | -13.22 | -25.74 to -0.6986 | * | 0.0329 |
| Saline vs. Whole cell culture (P3) / Carrageenan | -25.53 | -38.05 to -13.01 | **** | < 0.0001 |
| Saline vs. CFS (P3) / Carrageenan | -36.31 | -48.83 to -23.79 | **** | < 0.0001 |
| Carrageenan vs. Indomethacin / Carrageenan | 40.66 | 28.14 to 53.18 | **** | < 0.0001 |
| Carrageenan vs. Whole cell culture (P3) / Carrageenan | 28.35 | 15.83 to 40.87 | **** | < 0.0001 |
| Carrageenan vs. CFS (P3) / Carrageenan | 17.57 | 5.049 to 30.09 | ** | 0.0015 |
| Indomethacin / Carrageenan vs. Whole cell culture (P3) / Carrageenan | -12.31 | -24.83 to 0.2114 | NS | 0.0565 |
| Indomethacin / Carrageenan vs. CFS (P3) / Carrageenan | -23.09 | -35.61 to -10.57 | **** | < 0.0001 |
| Whole cell culture (P3) / Carrageenan vs. CFS (P3) / Carrageenan | -10.78 | -23.30 to 1.741 | NS | 0.1273 |

^a^CI: Confidence interval, ^b^NS: Non-significant, * < 0.05, ** < 0.01, *** P < 0.001, **** P < 0.0001.
